# Commentary: Bibliometric and visual analysis in the field of macrophages in Traditional Chinese Medicine from 2003 to 2023

**DOI:** 10.3389/fimmu.2026.1838577

**Published:** 2026-08-12

**Authors:** Ge Zhu, Mingjie Xu, Jingxing Li, Jiachun Xu, Zhen Zhou

**Affiliations:** 1 Tianjin University of Traditional Chinese Medicine, Tianjin, China; 2 Acupuncture Center for Brain Diseases, The Second Affiliated Hospital of Tianjin University of Traditional Chinese Medicine, Tianjin, China

**Keywords:** bibliometrics, CiteSpace, macrophages, traditional chinese medicine, VOSviewer

## 1 Introduction

We read with great interest the article by Zhang et al., titled “Bibliometric and visual analysis in the field of macrophages in Traditional Chinese Medicine from 2003 to 2023,” published in Frontiers in Immunology (1). This study represents the first systematic bibliometric analysis of two decades of TCM–macrophage research, utilizing data from the Web of Science Core Collection (WoSCC) and visualization tools (R/bibliometrix, VOSviewer, and CiteSpace). We fully endorse and commend the authors’ efforts and achievements. However, a critical appraisal of the manuscript reveals several methodological limitations, inconsistencies in data reporting, and insufficient analytical depth.

First, the study suffers from inappropriate database and literature selection. Its principal limitation lies in the exclusive reliance on the Web of Science Core Collection (WoSCC), thereby excluding non-English publications, Chinese core databases (e.g., CNKI and Wanfang), and non-journal literature such as conference proceedings and theses. Although WoSCC is widely regarded as the gold standard for international bibliometric analyses, its coverage of Chinese-language TCM research is inherently limited, potentially introducing substantial coverage bias. Given that TCM is a China-led discipline, a considerable volume of high-quality macrophage-related research is published in Chinese core journals. Omitting these sources precludes a comprehensive portrayal of the field’s research landscape and significantly underrepresents the contributions of Chinese scholars and institutions. Admittedly, integrating data from heterogeneous databases (e.g., combining WoSCC and CNKI) within bibliometric software such as VOSviewer or CiteSpace presents significant technical challenges. These include resolving disparate citation formats, managing complex duplicate records (deduplication), and ensuring accurate data matching across different indexing standards. Despite the technical challenges of integrating data from different databases, the authors should have explicitly addressed these constraints or justified the exclusion of key regional sources. Meanwhile, the search strategy employed—topic-based (TS) queries combining “macrophage” with various TCM-related terms—may lack sufficient precision. While TS searches are commonly used, their broad scope risks retrieving articles in which these terms appear only peripherally rather than as central themes. A more refined approach, such as combining title (TI) and author keywords (AK) fields, would likely yield a more focused dataset, thereby enhancing the validity of subsequent co-occurrence and clustering analyses. Likewise, although the manuscript presents the final search query, it omits critical details regarding its development and refinement. Specifically, it does not clarify whether preliminary searches were conducted to optimize keyword selection, whether non-research items (e.g., conference abstracts, reviews, or corrections) were excluded, or whether strategies were implemented to account for synonyms and spelling variants. These omissions compromise both recall and precision of the literature retrieval and further undermine the study’s reproducibility. Furthermore, the WoSCC data may contain duplicate records (e.g., the same article indexed under multiple categories) and author name ambiguities. The manuscript does not specify whether author disambiguation, institutional normalization, or deduplication procedures were performed. This omission risks introducing bias into the dataset, potentially compromising the accuracy of collaboration network mapping and bibliometric impact assessments.

Second, the authors explicitly state that they excluded macrophage subsets—such as Kupffer cells and alveolar macrophages—to “avoid disease-specific bias.” However, this rationale is unsubstantiated. Tissue-resident macrophage subsets are key therapeutic targets of TCM in liver, respiratory, and gastrointestinal disorders. Their exclusion renders the analysis of TCM–macrophage interactions incomplete and overly generalized, substantially diminishing the study’s relevance to clinical and translational TCM research (2, 3).

Furthermore, the manuscript fails to report critical analytical parameters, rendering the study irreproducible. While the authors mention the use of R (bibliometrix), VOSviewer, and CiteSpace, they omit all essential configuration details—such as the minimum occurrence thresholds for keywords or institutions in VOSviewer, time-slice settings and node selection criteria (e.g., Top N or Top N%) in CiteSpace, and the specific definitions or algorithms used in R for calculating metrics like the H-index or centrality measures. Transparent reporting of these parameters is fundamental to the reproducibility of bibliometric research; their absence precludes independent validation or replication of the analytical workflow.

The Results section—the core output of any bibliometric study—suffers from methodological misapplication and subjective interpretation, leading to fragmented and unpersuasive findings. First, much of the reported results are limited to descriptive statistics without substantive interpretation. For instance, statements such as “China published 1,201 articles versus 88 from the United States” merely restate raw data without exploring underlying drivers. Notably, the study reports that mainland China’s average citation per paper (25.5) is substantially lower than those of the United States (50.02), Australia (48.27), and even Taiwan (30.6), yet offers no analysis of potential causes—such as language barriers, limited international collaboration, or a predominance of basic over clinical research. Similarly, while the low rate of international collaboration among Chinese authors (8.3%) is noted, the structural or systemic factors contributing to this are not examined. Second, the keyword co-occurrence network generated in VOSviewer is divided into four clusters, but the analysis stops at listing representative terms per cluster. Crucially, it lacks temporal integration—no examination of keyword dynamics, thematic evolution, or shifting research foci over time is provided. Moreover, the clustering appears to rely solely on manual labeling, with no quantitative metrics (e.g., silhouette scores, modularity values) reported to validate cluster robustness or coherence. Third, the study presents bibliometric dimensions—publication trends, country/region contributions, institutional output, author productivity, journal co-citation, and keyword analysis—as isolated components, failing to synthesize them into an integrated knowledge map. This fragmented presentation lacks a unifying analytical framework. Given the 20-year study span, the exclusive use of cumulative metrics (e.g., total publications, total citations, static rankings) overlooks the temporal capabilities of tools like CiteSpace and VOSviewer. Key dynamic features—such as burst detection, thematic evolution maps, or timeline visualizations—that could reveal how research frontiers have shifted over time remain unexploited. Finally, the manuscript concludes that liver disease, colitis, and cancer represent emerging research frontiers. However, this assertion is not grounded in bibliometric evidence—such as strong citation bursts, high betweenness centrality, or recent high-impact publications—but instead reads as speculative inference, lacking the methodological rigor expected in bibliometric forecasting.

Finally, we reiterate our appreciation and commendation for the valuable work by Zhang et al., which not only offers strategic insights for researchers but also helps chart a course for future studies on Traditional Chinese Medicine and macrophage biology. Nevertheless, to uphold the rigor and scientific integrity of the manuscript, more precise methodological reporting and cautious interpretation of findings are warranted.
